# Application of luteolin nanomicelles anti-glioma effect with improvement *in vitro* and *in vivo*

**DOI:** 10.18632/oncotarget.18019

**Published:** 2017-05-19

**Authors:** Songping Zheng, Yongzhong Cheng, Yan Teng, Xiaoxiao Liu, Ting Yu, Yi Wang, Jiagang Liu, Yuzhu Hu, Cong Wu, Xiang Wang, Yanhui Liu, Chao You, Xiang Gao, Yuquan Wei

**Affiliations:** ^1^ Department of Neurosurgery and Institute of Neurosurgery, State Key Laboratory of Biotherapy, West China Hospital, West China Medical School, Sichuan University and Collaborative Innovation Center for Biotherapy, Chengdu, 610041, PR China

**Keywords:** luteolin, glioma, chemotherapy, nanoparticles

## Abstract

Glioblastoma multiforme (GBM) is one of the most common and malignant tumor. Luteolin, a polyphenolic compound, has been proposed to have anti-tumor activity against various cancers. However, the greatest obstacle in the administration of luteolin is its hydrophobicity as well as the low oral bioavailability. In this study, we formulated luteolin-loaded MPEG-PCL (Luteolin/MPEG-PCL) micelles aiming to improve its solubility in aqueous solution and investigate the anti-tumor effect on glioma *in vitro* and *in vivo*. The spherical Luteolin/MPEG-PCL micelles were completely dispersible in normal saline and could release luteolin in a sustained manner *in vitro*. We demonstrated that Luteolin/MPEG-PCL micelles had stronger cytotoxicity and induced a higher percentage of apoptosis in C6 and U87 cells than free luteolin *in vitro*. The immunohistochemical study revealed that Luteolin/MPEG-PCL micelles induced more glioma cell apoptosis than free luteolin and inhibited neovascularization in tumor tissues. The Pro-caspase9 and Bcl-2 down-regulation and cleaved-caspase9 and Bax up-regulation suggested that luteolin induced apoptosis via the mitochondrial pathway *in vitro*. What is more, we found the drug could cumulated much more in the nano-drug group than free drug group through imaging *in vivo*. In conclusion, the Luteolin/MPEG-PCL micelles have the potential clinical application in glioma chemotherapy.

## INTRODUCTION

Glioma is the most common primary malignant brain tumor in children and adults, and has been notoriously known to be a great challenge for clinical treatment. Glioblastoma (GBM), which is classified as Grade IV by the World Health Organization (WHO), is the most aggressive and deadly subtype and accounts for more than 50 percent of glioma [1]. With the development of neuroimaging, GMB can be early diagnosed and more details could be obtained from MRI. However, treated with comprehensive therapy which consists of surgical resection followed by radiotherapy and chemotherapy, the median survival time of patients with GBM is only 15-17 months [2–4].

GMB features a high proliferation activity and a diffuse nature having a tendency to invade surrounding brain tissues. Due to several factors, the effect of conventional chemotherapy is unsatisfactory. First, the effective accumulation of chemotherapy agents in the central nervous system is limited because of the blockage of blood–brain barrier (BBB). Secondly, recent research showed that glioma cells over-express the multi-drug resistance (MDR) transporters which lead to the extruding of agents resulting in the phenomenon of chemoresistance [5]. Moreover, glioma cells are able to survive in extreme conditions such as glucose deprivation or stress by autophagy or modulating multiple signaling pathways [6–9]. Hence, more effective chemotherapy drugs are sorely in need.

Luteolin is an important natural polyphenolic compound, which mainly derived from diverse fruits and vegetables, including pomegranate, cabbage, celery, spinach and so forth. It has been widely concerned and researched extensively due to its diverse pharmacological effects on anti-virus, anti-inflammatory and anti-tumor [10–14]. Additionally, with the dietary supplement of luteolin, the diet-induced insulin resistance in mice was obviously ameliorated [15, 16]. Studies have confirmed its effectiveness in suppressing a wide variety of human tumor, including colorectal cancer, cervical cancer, lung cancer, skin cancer, etc [12, 14, 17–19]. Compared with conventional chemotherapy agents, luteolin possesses a prominent advantage of low toxicity. It has been reported that luteolin selectively acts on tumor cells and normal cells are not affected [20]. Another strong evidence demonstrating the safety of the agent is the widespread use of luteolin in diet by millions of people in many countries. However, the clinical application of luteolin is hindered by its intrinsic poor water-solubility as well as the low oral bioavailability.

The utilization of nanomaterials in cancer treatment is of intriguing interest. Different nanomaterials such as carbon, metal, or lipid-based nanoparticles are of diverse sizes, structures, physicochemical properties, surface properties and biocompatibilities, so as to meet specific requirements.

Various nanomaterials have been formulated to deliver chemotherapy agents to tumor sites due to highly hydrophobic nature of these agents. We have previously reported the efficiently delivery of polyphenolic compound by the biodegradable monomethyl poly(ethylene glycol)-poly(ε-caprolactone) (MPEG-PCL) micelles in cancer therapy [21]. After encapsulation, the hydrophobic core part of MPEG-PCL micelles functions as a container of agents while the hydrophilic shell part greatly increases its solubility in aqueous media. In this research, luteolin loaded MPEG-PCL (Luteolin/MPEG-PCL) micelles were prepared by a self-assembly method. The morphology, zeta potential, encapsulation efficiency and pharmacokinetics of Luteolin/MPEG-PCL micelles were investigated. In the next step, the anti-tumor activity of Luteolin/MPEG-PCL micelles was evaluated *in vitro* and *vivo* (nude mice and zebrafish).

## RESULTS

### Preparation and characterization of Luteolin/MPEG-PCL micelles

**Figure 1:**
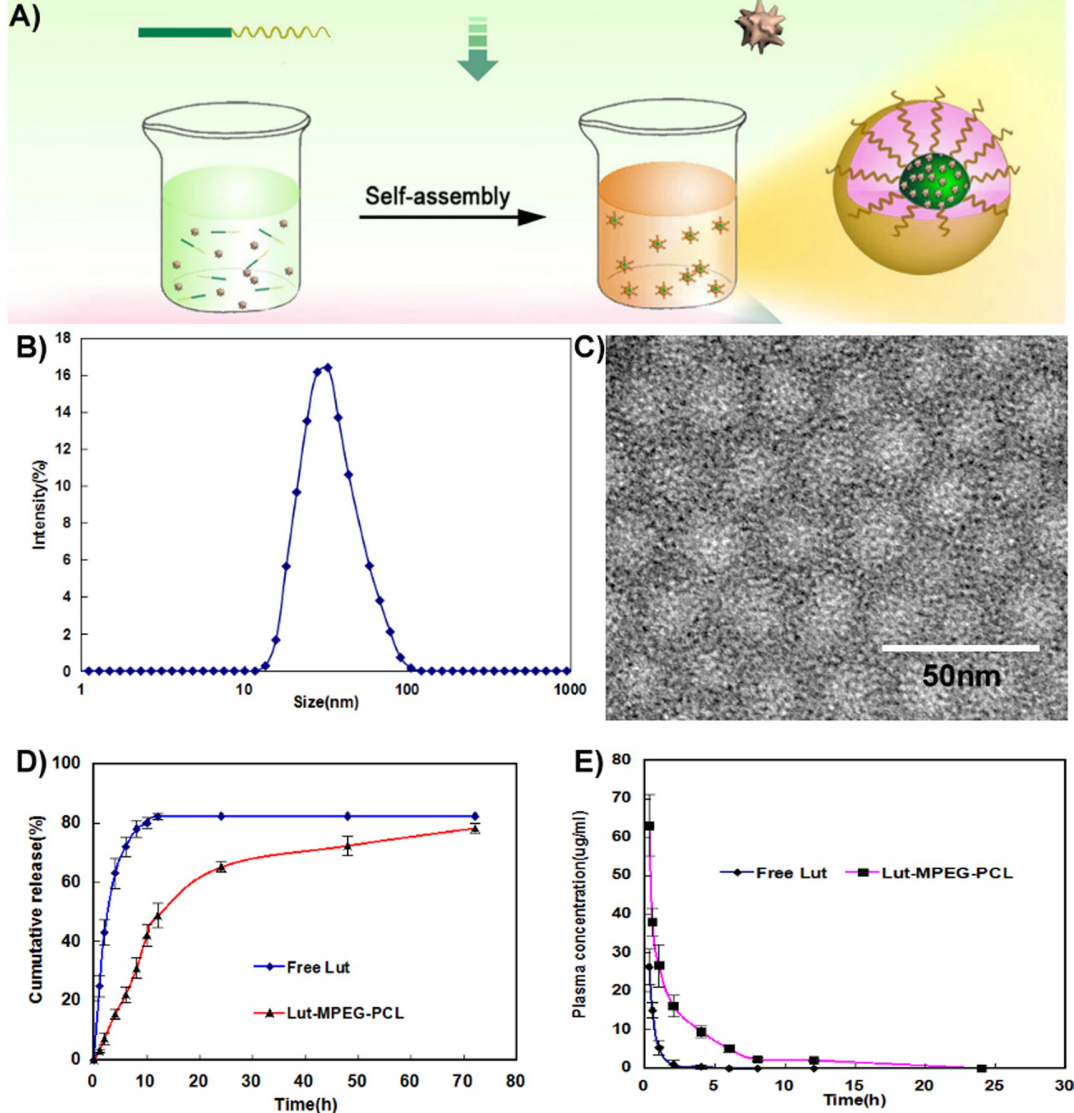
Preparation and identification of Luteolin/MPEG-PCL micelles **(A)** Preparation of Luteolin/MPEG-PCL micelles. Luteolin/MPEG-PCL micelles were prepared by self-assembly methods. First, luteolin and MPEG-PCL were co-dissolved in acetone. Then the mixture was added into water, and in this process luteolin and MPEG-PCL assembled into micelles with luteolin and PCL inside and PEG outside. **(B)** The size of Luteolin/MPEG-PCL micelles. **(C)** Transmission electron microscopy(TEM) image of Luteolin/MPEG-PCL micelles; **(D)** Release of free luteolin and Luteolin/MPEG-PCL micelles *in vitro*; **(E)** Pharmacokinetics of free luteolin and Luteolin/MPEG-PCL micelles.

Luteolin/MPEG-PCL micelles were synthesized by a single-step precipitation method, as previously reported [21]. Luteolin/MPEG-PCL micelles contains a ball-shaped hydrophilic PEG shell and a hydrophobic PCL core where luteolin was encapsulated into the core part of micelles and surrounded with PEG to improve its water solubility (Figure 1A).

The DL and EE of Luteolin/MPEG-PCL micelles were 6% and 98.5%, respectively. As shown in Figure 1B, the particle size of Luteolin/MPEG-PCL micelles measured by DLS was 36.5 nm (PDI=0.12). Moreover, the size was confirmed by TEM (Figure 1C), which was consistent with the DLS results. Since Luteolin/MPEG-PCL micelles measured by DLS was in aqueous phase and structure of these amphiphilic particles was usually loose in solution, the DLS size was always larger compared to TEM size. The surface of Luteolin/MPEG-PCL micelles was negatively charged, with the zeta potential of -3.2 mv.

The *in vitro* release profile of free luteolin and luteolin from Luteolin/MPEG-PCL micelles was examined in PBS (PH7.4) at 37 °C. As shown in Figure 1D, free luteolin exhibited a rapid release and the accumulated release reached a peak of 79.2%±1.9% in 12h. In comparison, about 46% luteolin was released from nano-micelles in the first 10h and another 34% of drug was released sustainedly over 70h. The cumulative curve indicated that the drug-release process of Luteolin/MPEG-PCL micelles was stable and sustained *in vitro* (p < 0.01, Luteolin/MPEG-PCL micelles versus free luteolin).

Concentration–time profiles for free luteolin and Luteolin/MPEG-PCL micelles after intravenous injection are shown in Figure 1E. The T_max_, T_1/2_, C_max_ and AUC of free luteolin were 15 min, 0.7 h, 27.32 μg/ml and 14.76 mg L^−1^ h^−1^ respectively, while the T_max_, T_1/2_, C_max_ and AUC of Luteolin/MPEG-PCL micelles were 15 min, 0.9 h, 63.27 μg/ml and 106.7 mg L^−1^ h^−1^ (p < 0.01, Luteolin/MPEG-PCL micelles versus free luteolin) respectively suggesting that MPEG-PCL micelles could improve the bioavailability and pharmacokinetics of luteolin *in vivo*.

### Anti-glioma effect *in vitro*

#### Luteolin suppressed cell viability and proliferation

**Figure 2:**
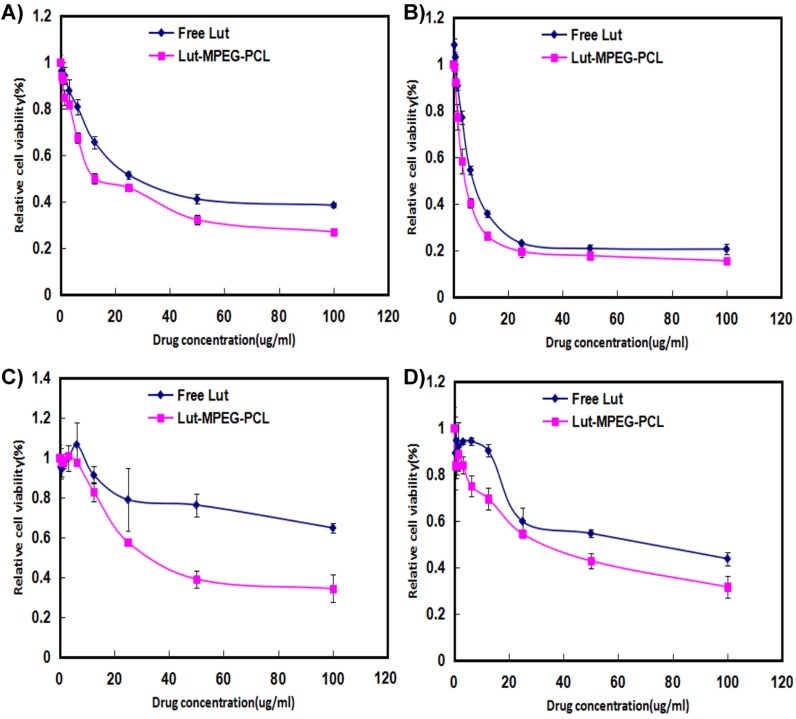
Cell viability detection with MTT methods Cells were treated with increasing concentrations of luteolin for 24 h and 48 h. Cell survival was measured by MTT. The MTT assay (Mean±SEM, n=6) showed that the Luteolin/MPEG-PCL micelles were more effective than free luteolin at concentrations of 6.25 μg/mL, 12.5 μg/mL, 25μg/mL, 50 μg/mL, and 100 μg/mL at 24 h **(A** and **C)** or 48 h **(B** and **D)** in the C6 **(A** and **B)** cell line and U87 **(C** and **D)** cell line.

The cytotoxicity of free luteolin and Luteolin/MPEG-PCL micelles was studied by MTT assay using C6 and U87 cells. As demonstrated in Figure 2, both free luteolin and Luteolin/MPEG-PCL micelles inhibited C6 (Figure 2A and Figure 2B) and U87 (Figure 2C and Figure 2D) proliferation in a time- and dose-dependent manner. With the drug concentration of Luteolin/MPEG-PCL micelles increasing from 0.4 μg/ml to 100 μg/ml, the relative cell viability of C6 decreased from 94% to 27% at 24h and 98% to 15.5% at 48h (Figure 2D), while that of U87 decreased from 99% to 34.2% at 24h and 83% to 31.5% at 48h (Figure 2B). Meanwhile, the half maximal inhibitory concentration (IC50) of Luteolin/MPEG-PCL micelles (C6: 12.5 μg/ml; U87: 30 μg/ml) was lower than that of free luteolin (C6: 46.3 μg/ml; U87: >100 μg/ml) at 24h (Figure 2A and Figure 2C) (p < 0.01, Luteolin/MPEG-PCL micelles versus free luteolin). The results revealed that Luteolin/MPEG-PCL micelles had cytotoxicity effect on glioma cells and the encapsulation of luteolin in MPEG-PCL micelles improved its cytotoxic activity.

**Figure 3:**
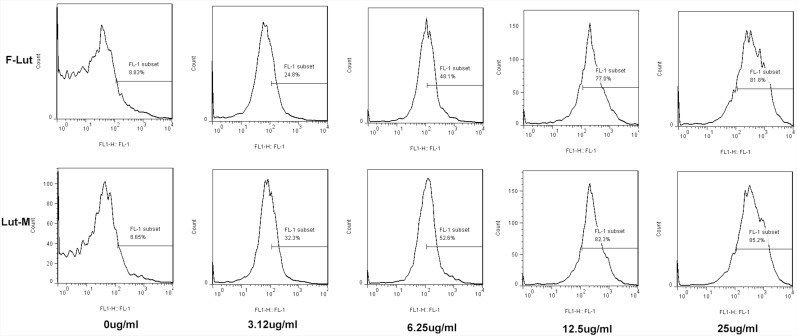
Cell proliferation determination with CFSE staining U87 cells were stained with CFSE and planted into six-well plates. On the second day, the drugs were added into cell medium. The cells were collected and detected by FCM after 48h. We found that Luteolin/MPEG-PCL micelles inhibited more cell proliferation than free drug.

Cell proliferation was detected by CFSE (Figure 3). We found that Luteolin/MPEG-PCL micelles inhibit 85.2%, 82.3%, 52.6% and 32.3% U87 cell proliferation at the concentration of 25 μg/ml, 12.5 μg/ml, 6.25 μg/ml and 3.125 μg/ml, respectively. But free luteolin only inhibit 81.8%, 77%, 48.1% and 24.8% at the same drug concentrations (p < 0.01, Luteolin/MPEG-PCL micelles versus free luteolin). And the results showed that Luteolin/MPEG-PCL could inhibit more U87 cells growth than free luteolin.

### Apoptosis induced by luteolin

**Figure 4:**
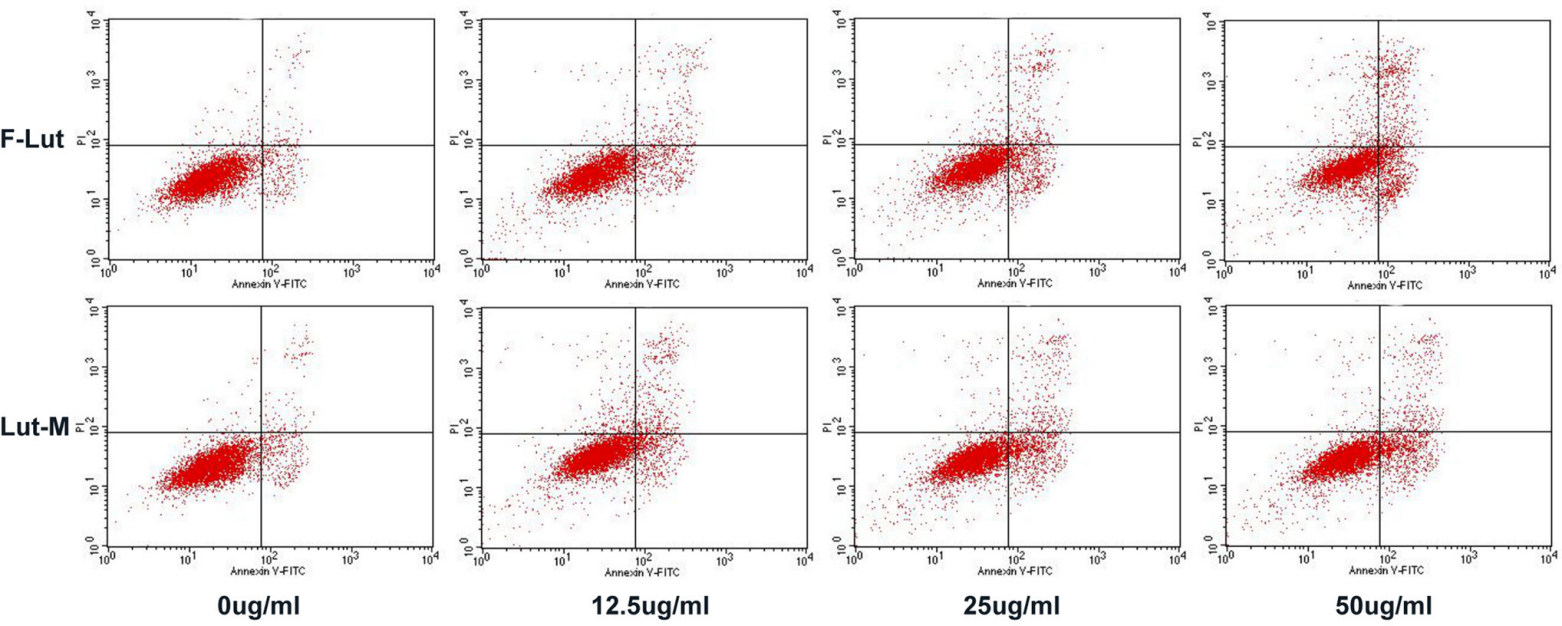
Cell apoptosis test with FCM U87 cells were planted into six-well plates. On the second day free luteolin and Lut/MPEG-PCL micelles were added into the medium and incubated with cells for 48 hours. At last, the cells were collected and stained with PI and Annexin V. Then, the cell apoptosis was analyzed by FCM.

As apoptosis is an important mechanism of anti-cancer chemotherapy, we detected cell apoptosis of U87 cells induced by free luteolin and Luteolin/MPEG-PCL micelles with annexin V-FITC and PI apoptosis assay. The ability of free luteolin and Luteolin/MPEG-PCL micelles to induce apoptosis of U87 cells is shown in Figure 4. In the NS and Blank micelle group, the apoptosis rate was 5.02% and 4.83%, respectively. In the free luteolin and Luteolin/MPEG-PCL micelle group, the apoptotic cells increased in a concentration-dependent manner. The apoptosis rates were 11.2% and 14.02%, 14.3% and 17.4%, 19.8 and 32.3% (p < 0.01, Luteolin/MPEG-PCL micelles versus free luteolin) when the U87 cells were treated with free luteolin and Luteolin/MPEG-PCL micelles at a concentration of 6.25 μg/ml, 12.5 μg/ml and 50 μg/ml, respectively. It is evident that Luteolin/MPEG-PCL micelle was capable of inducing more U87 cell apoptosis than other groups.

### Western blotting

**Figure 5:**
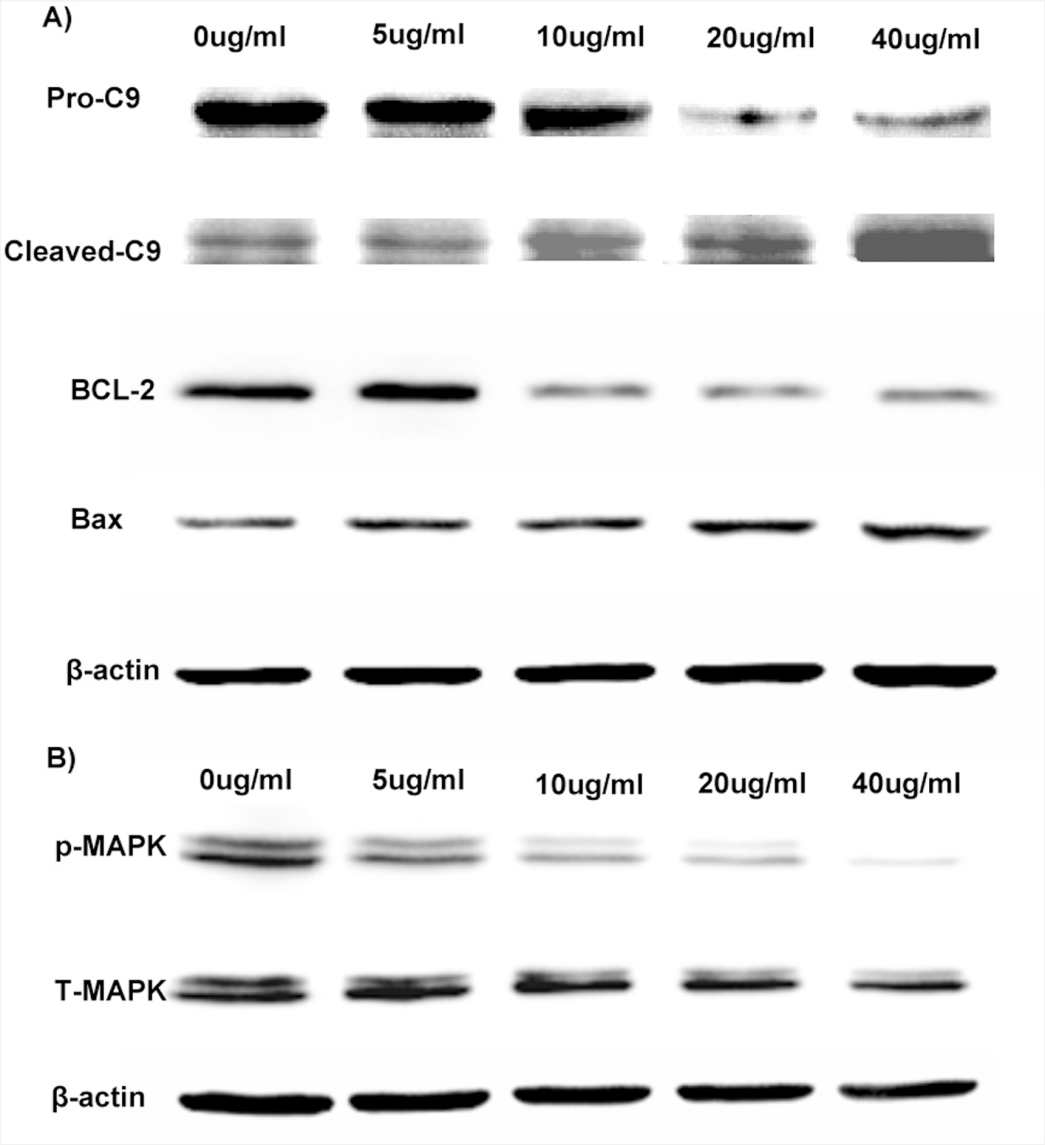
Anti-glioma molecular mechanism of Luteolin/MPEG-PCL micelles U87 cells were exposed to Luteolin/MPEG-PCL micelles for 48 h and cell extract proteins subjected to Western blotting analysis. **(A)** anti-apoptotic protein Bcl-2 was down-regulated and proapoptotic protein Bax was up-regulated. Luteolin/MPEG-PCL micelles induced glioma cell apoptosis through down-regulating anti-apoptotic protein Bcl-2 and up-regulating proapoptotic protein Bax and triggering the caspase-9. **(B)** p-MAPK was down-regulated but total MAPK was not changed. Luteolin/MPEG-PCL micelles inhibited glioma cell proliferation through down-regulating p-MAPK.

To further investigate the underline mechanism of the anti-cancer effect of Luteolin, gene expressions related to cell apoptosis and proliferation following treatment of free luteolin and Luteolin/MPEG-PCL micelles were examined. As demonstrated in Figure 5A, the Bcl-2 protein expression was decreased while the protein expression of Bax increased after treatment of free luteolin and Luteolin/MPEG-PCL micelles, indicating that luteolin induced apoptosis through the mitochondrial pathway. As demonstrated in Figure 5B, the levels of MAPK and p-MAPK after luteolin treatment were also examined showing obviously decreased p-MAPK after 48h treatment of Luteolin/MPEG-PCL micelles, whereas the total MAPK were unchanged.

### Anti-glioma effect *in vivo*

#### Luteolin inhibited tumor growth in animal models

**Figure 6:**
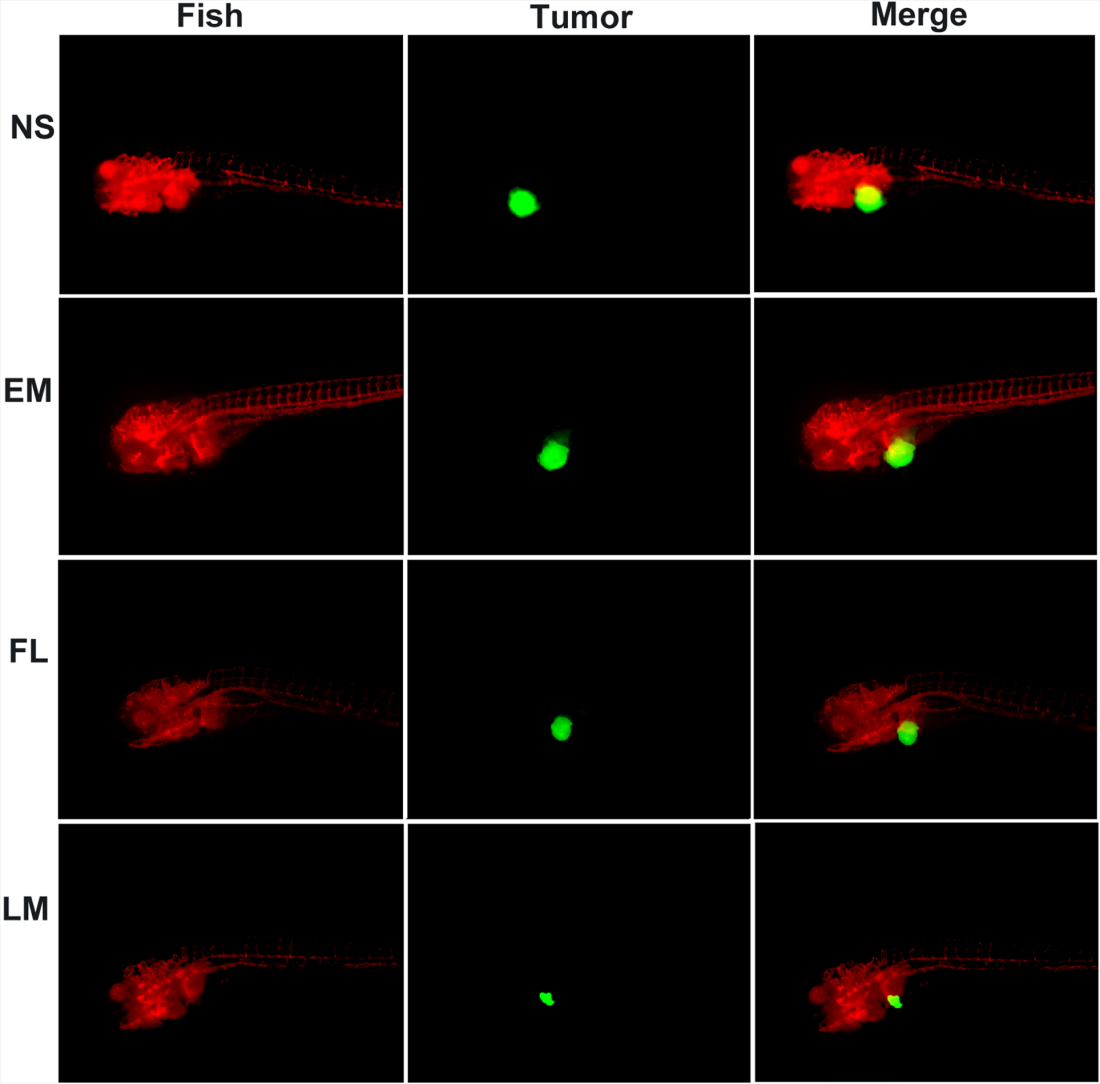
Anti-glioma effect of luteolin and Luteolin/MPEG-PCL micelles in zebrafish model Zebrafish were injected with 300 tumor cells into the perivitelline space using a Cell Tram Vario injector with a glass micropipette. At 24 h post-implantation, tumors from zebrafish treated with NS, empty micelles (EM), FL, or LM micelles are shown. The Lut/MPEG-PCL micelles inhibited tumor growth in zebrafish more effectively than other groups.

The transgenic zebrafish model and nude mice xenograft model were employed to evaluate the anti-glioma effect of free luteolin and Luteolin/MPEG-PCL micelles. Three days after injection of perivitelline into the zebrafish, U87 cells formed a solid tumor (green fluorescence). Shown in Figure 6, antitumor effects were observed in FL, and LM nano-micelles groups when compared with the control and blank micelles group. As detected by confocal microscopy, the tumors in the Luteolin/MPEG-PCL micelles group were smaller than FL group. No antitumor effects was observed in blank micelles group. The average tumor volume in the LM nano-micelles group was significantly smaller than that of NS, EM and FL groups. These data indicated that MPEG-PCL could enhance the anti-glioma cancer activity of luteolin *in vivo*.

**Figure 7:**
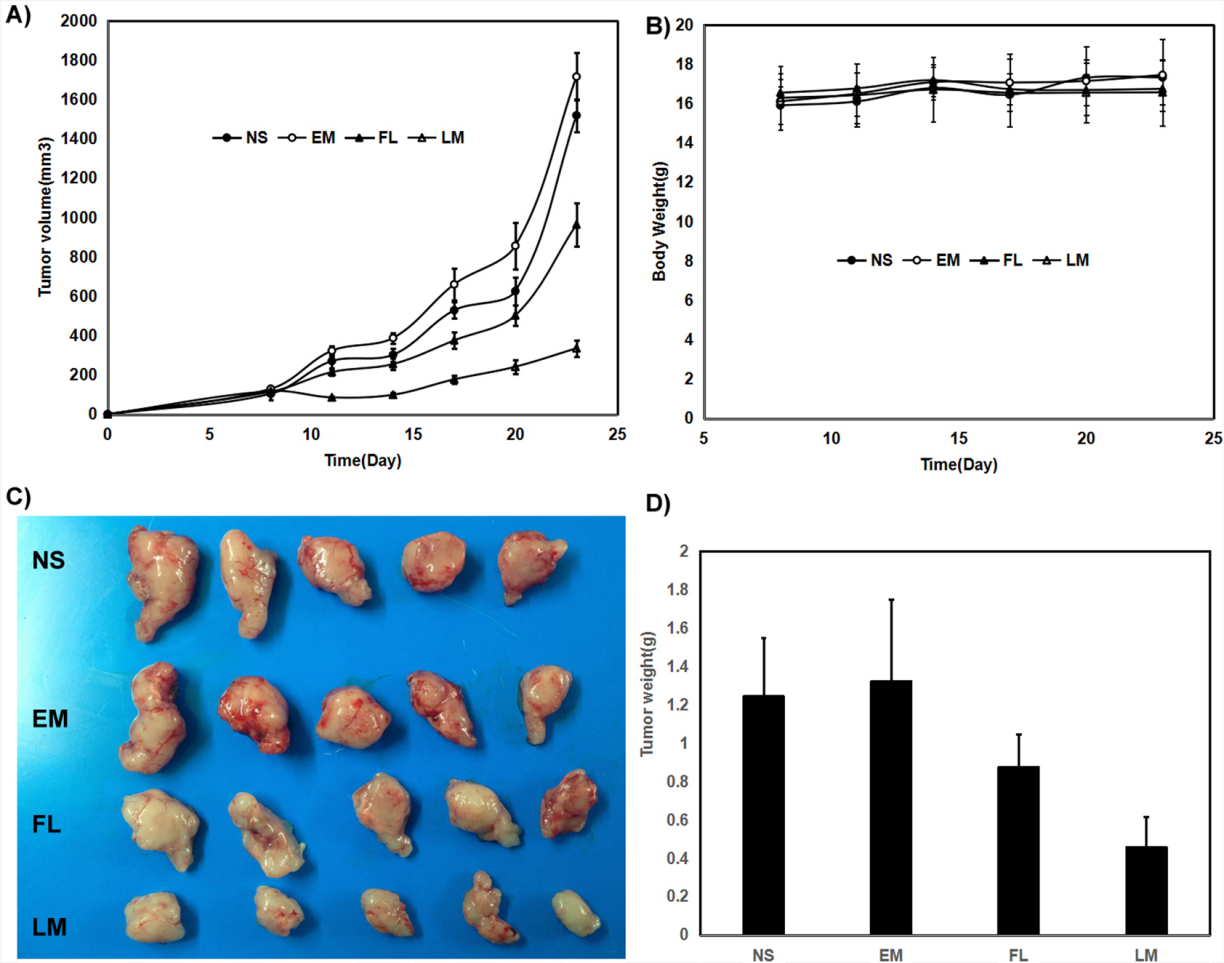
Anti rat glioma C6 effect of luteolin and Luteolin/MPEG-PCL micelles in nude mouse model Female nude mice were inoculated with C6 cells on day 0. On day 7, the mice were randomized into four groups, and were injected intravenously with saline (NS), empty MPEG-PCL micelles (EM), free luteolin (FL), or Luteolin/MPEG-PCL micelles (LM). **(A)** Tumor growth curves. **(B)** Body weight of different groups. **(C)** Tumor photos of NS, DMP, DMP-pVax and DMP-pIL12 treatment groups. **(D)** Tumor weight. (Mean±SEM, n=5) (p<0.01, LM versus NS, EM, FL, A; LM versus NS, EM, FL, D).

In mice bearing C6 subcutaneous glioma model, the blank micelles had no anti-glioma effects. The tumor volumes decreased by 40% and 78% respectively under exposure of free luteolin and Luteolin/MPEG-PCL micelles in C6 glioma model with the same interventions in the Figure 7A (p < 0.01, Luteolin/MPEG-PCL micelles versus free luteolin). Free luteolin and Luteolin/MPEG-PCL micelles exerted significant anti-giloma effects without obviously impact of mice body weight in the Figure 7B. And the representative photographs of subcutaneous gliomas were shown in Figure 7C. The tumor weights in the Luteolin/MPEG-PCL micelle group were lower than that of other groups in both animal models as well in the Figure 7D. The tumor weights in the Luteolin/MPEG-PCL micelle group were lower than that of other groups in the Figure 7D (p < 0.01, Luteolin/MPEG-PCL micelles versus free luteolin).

**Figure 8:**
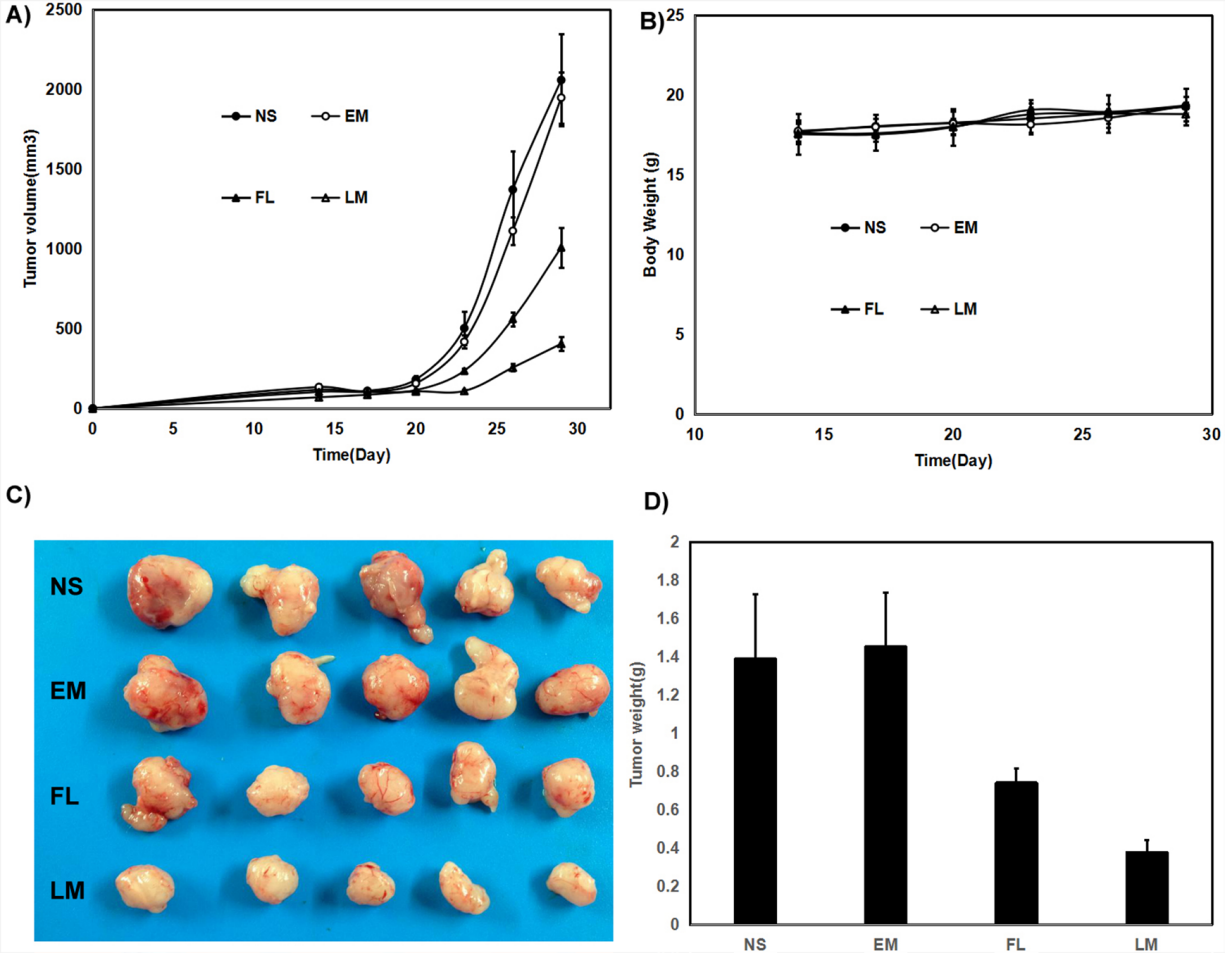
Anti-human glioma U87 effect of luteolin and Luteolin/MPEG-PCL micelles in nude mouse model Female nude mice were inoculated with U87 cells on day 0. On day 14, the mice were randomized into four groups, and were injected intravenously with saline (NS), empty MPEG-PCL micelles (EM), free luteolin (FL), or Luteolin/MPEG-PCL micelles (LM). **(A)** Tumor growth curves. **(B)** Body weight of different groups. **(C)** Tumor photos of NS, DMP, DMP-pVax and DMP-pIL12 treatment groups. **(D)** Tumor weight. (Mean±SEM, n=5) (p<0.01, LM versus NS, EM, FL, A; LM versus NS, EM, FL, D).

In mice bearing U87 subcutaneous glioma model, the tumor volumes decreased by 48.7% and 79.5% respectively under exposure of free luteolin and Luteolin/MPEG-PCL micelles in U87 glioma model with the same interventions in the Figure 8A (p < 0.01, Luteolin/MPEG-PCL micelles versus free luteolin). Free luteolin and Luteolin/MPEG-PCL micelles exerted significant anti-giloma effects without obviously impact of mice body weight in the Figure 8B. And the representative photographs of subcutaneous gliomas were shown in Figure 8C. The tumor weights in the Luteolin/MPEG-PCL micelle group were lower than that of other groups in both animal models as well in the Figure 8D. The tumor weights in the Luteolin/MPEG-PCL micelle group were lower than that of other groups in the Figure 8D (p < 0.01, Luteolin/MPEG-PCL micelles versus free luteolin).

The results of both animal models were consistent and the representative photographs of subcutaneous gliomas were shown in Figure 7 and Figure 8. The tumor weights in the Luteolin/MPEG-PCL micelle group were lower than that of other groups in both animal models as well. It suggested that the anti-glioma activity of luteolin was enhanced with the help of MPEG-PCL micelles delivery system.

### Histological analysis by TUNEL assay, Ki67 and CD31

**Figure 9:**
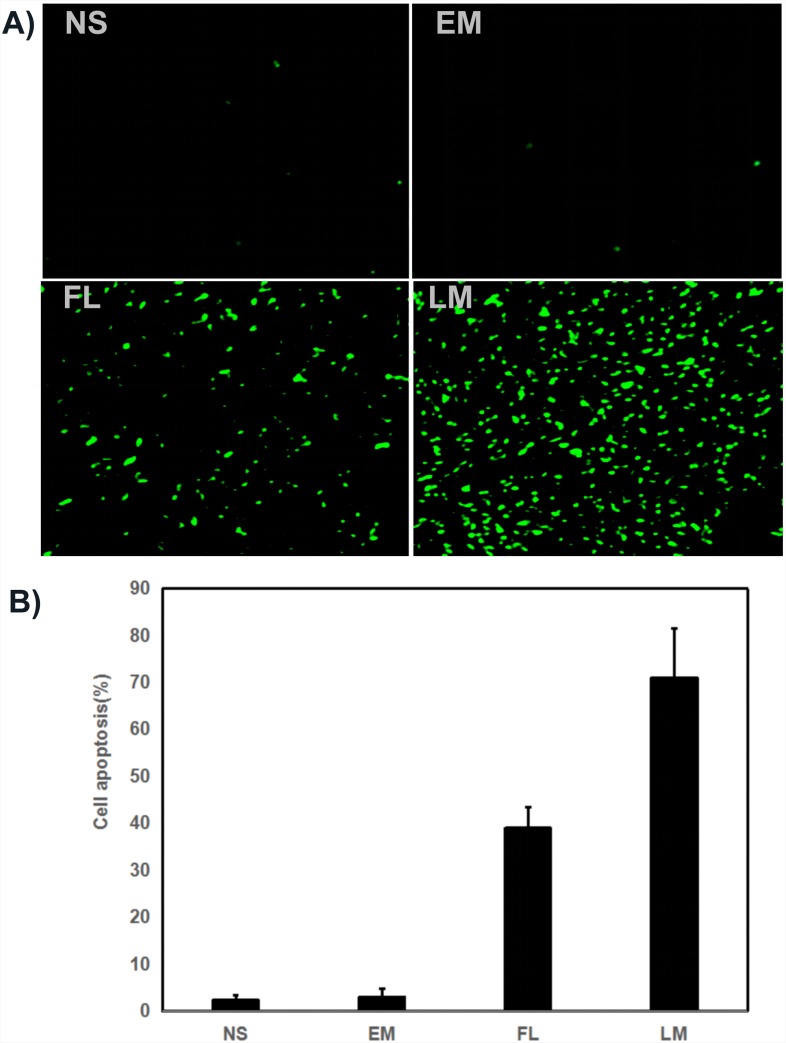
Tunnel test **(A)** Cell apoptosis was assessed by counting the number of TUNEL-positive cells in the field, and Luteolin/MPEG-PCL micelles(LM) was superior to other controls in increasing cell apoptosis. LM significantly increased more cell apoptosis than other groups. **(B)** The stained TUNEL positive cells were counted in five high power fields (Mean±SEM, n=5, p<0.01, LM versus NS, EM, LM, B).

Tumor sections were stained with TUNEL for the investigation of cell apoptosis in four groups, which were treated with NS, blank micelles, free luteolin and Luteolin/MPEG-PCL micelles respectively. As shown in Figure 9, no considerable positve nuclei was found in the NS and blank micelles group while substantial amount of TUNEL stains were observed in the Luteolin/MPEG-PCL micelle group. The apoptotic index was 2.3% ±1.2% in the NS group, 3% ±1.73% in the blank micelles group, 39% ±4.4% in the free luteolin group (p < 0.01, free luteolin versus NS and blank micelles) and 71% ±10.5% in the Luteolin/MPEG-PCL micelle group (p < 0.01, Luteolin/MPEG-PCL micelles versus free luteolin, NS and blank micelle). The results indicated that luteolin encapsulated in the MPEG-PCL micelles was more capable of inducing apoptosis of U87 cells *in vivo*.

**Figure 10:**
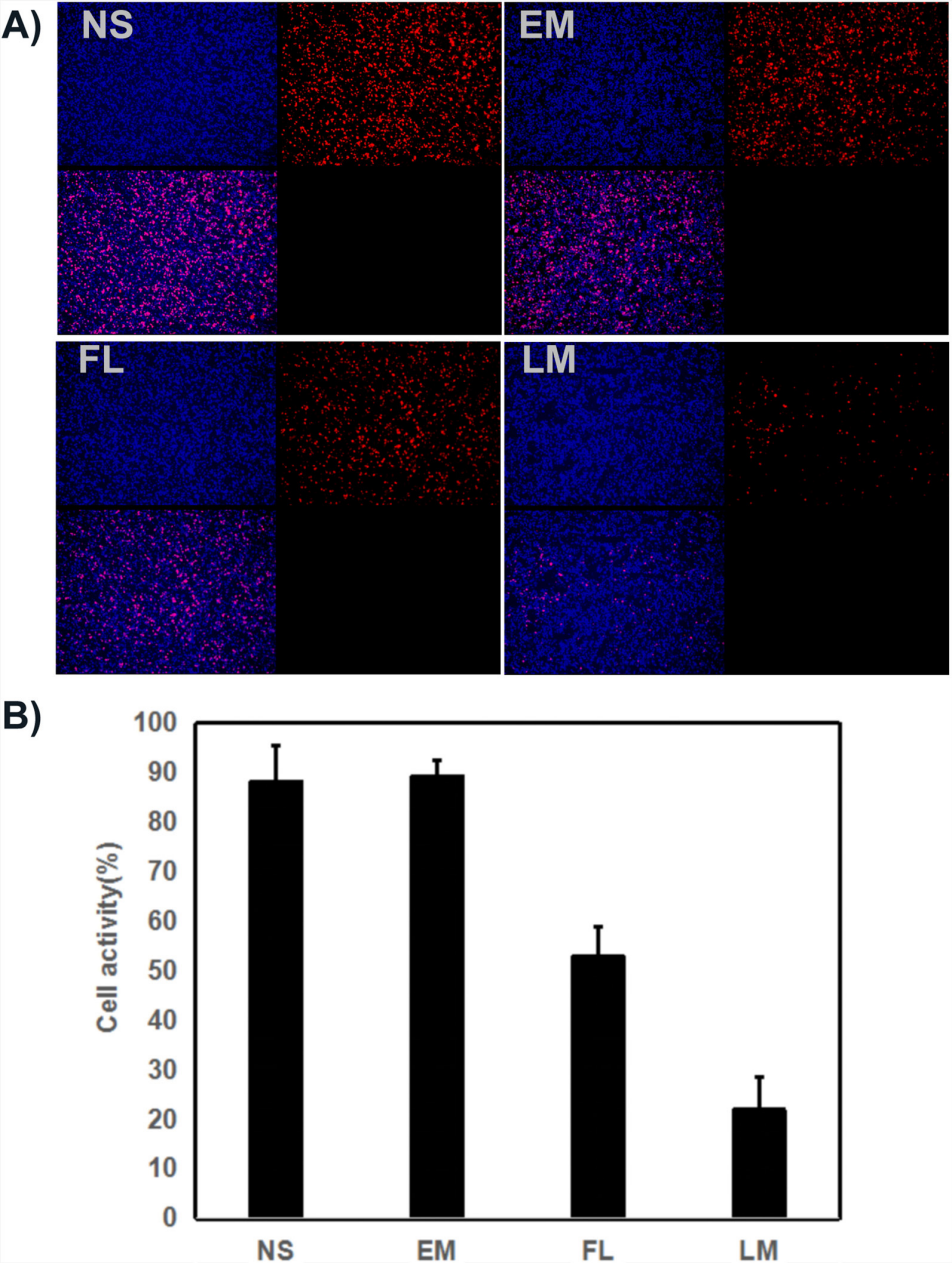
Cell proliferation test with ki67 staining **(A)** Cell proliferation was assessed by counting the number of ki67-positive cells in the field, and Luteolin/MPEG-PCL micelles(LM) inhibited more cell proliferation than other groups. **(B)** The stained ki67 positive cells were counted in five high power fields (Mean±SEM, n=5, p<0.01, LM versus NS, EM, LM, B).

The U87 cell proliferation was evaluated by immunohistochemical staining. Tumor sections from four groups were stained with Ki-67. As shown in Figure 10, the positive cells which emitted red fluorescence were abundant in the NS group, blank micelle group and free luteolin group. In comparison, few positive cells were observed in the Luteolin/MPEG-PCL micelle group. The Ki-67 labelling index, which is usually related to the clinical course of tumor, is significantly lower in Luteolin/MPEG-PCL micelle group (22% ±6.5%) than other groups (88% ±7.2% in NS group, 89.3% ±3.2% in blank micelle group, 53% ±6% in free luteolin group) (p < 0.01, Luteolin/MPEG-PCL micelles versus free luteolin, NS and blank micelle). It is evident that Luteolin/MPEG-PCL micelles effectively suppressed tumor proliferation.

**Figure 11:**
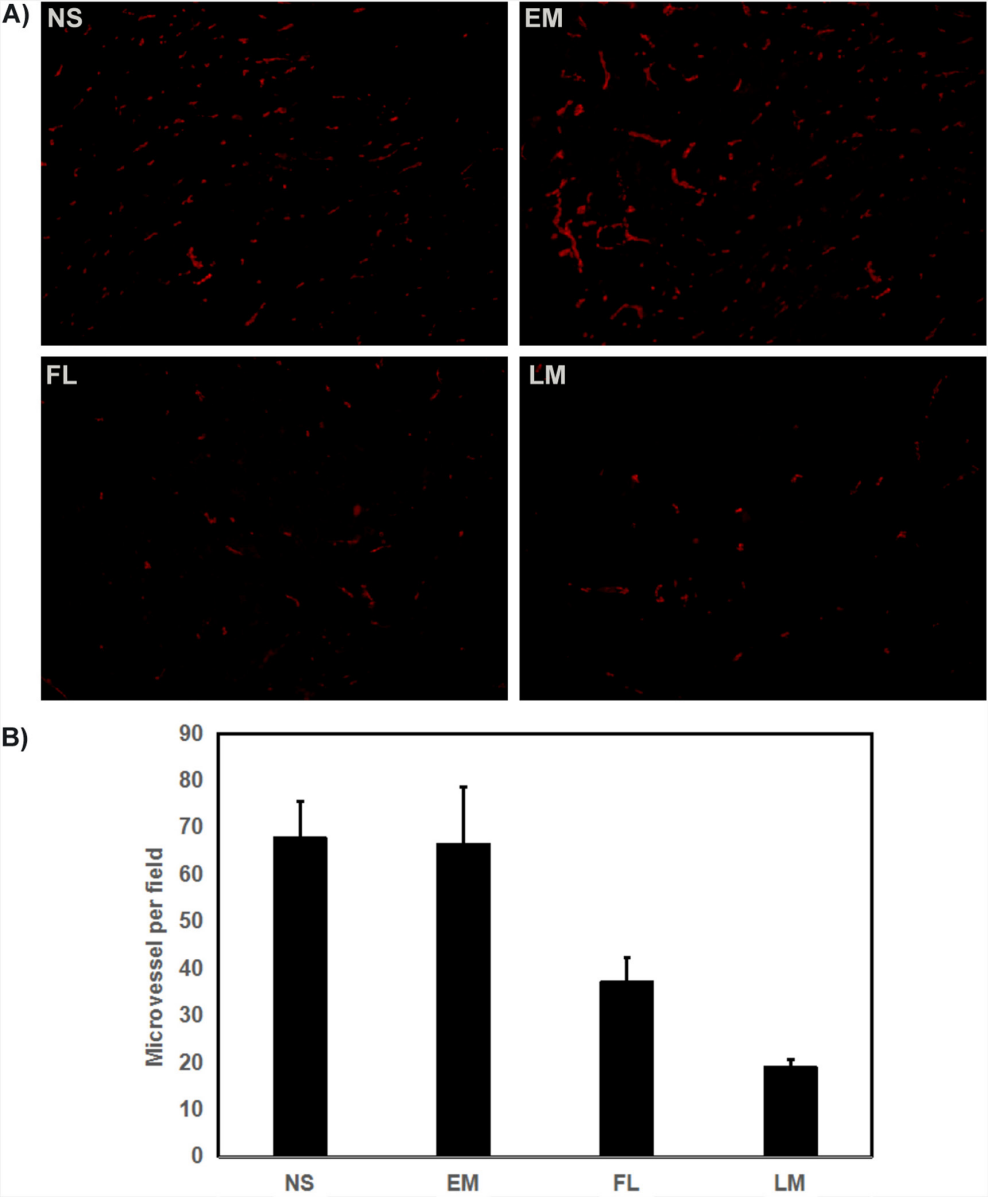
Tumor sections of each treatment group were stained with CD31 for evaluating the microvessel density (MVD) Tumor tissues were collected after the final treatment and tumor sections of NS, EM, FL, and LM were stained with CD31 antibody **(A)**. The CD31 assay vascular number (Mean±SEM, n=5) **(B)**. The LM treatment caused inhibition of angiogenesis in the tumors. This implies that anti-angiogenic effect may be a mechanism of inhibiting U87 cells by FL and LM *in vivo*, and that LM inhibited more angiogenesis than other groups.

Furthermore, the angiogenesis of U87 glioma was explored as well. According to Figure 11, few microvessels with red fluorescence were observed group treated with Luteolin/MPEG-PCL micelles. The number of microvessels was significantly lower in the Luteolin/MPEG-PCL micelle group (19±2.2) compared with NS group (67.8±7.8), blank micelle group (66.4±12.2) and free luteolin group (37.2±5.16) (p < 0.01, Luteolin/MPEG-PCL micelles versus free luteolin, NS and blank micelle). We propose that the mechanism of anti-angiogenesis may play an important role in inhibiting tumor growth by Luteolin/MPEG-PCL micelles.

### Imaging *in vivo*

**Figure 12:**
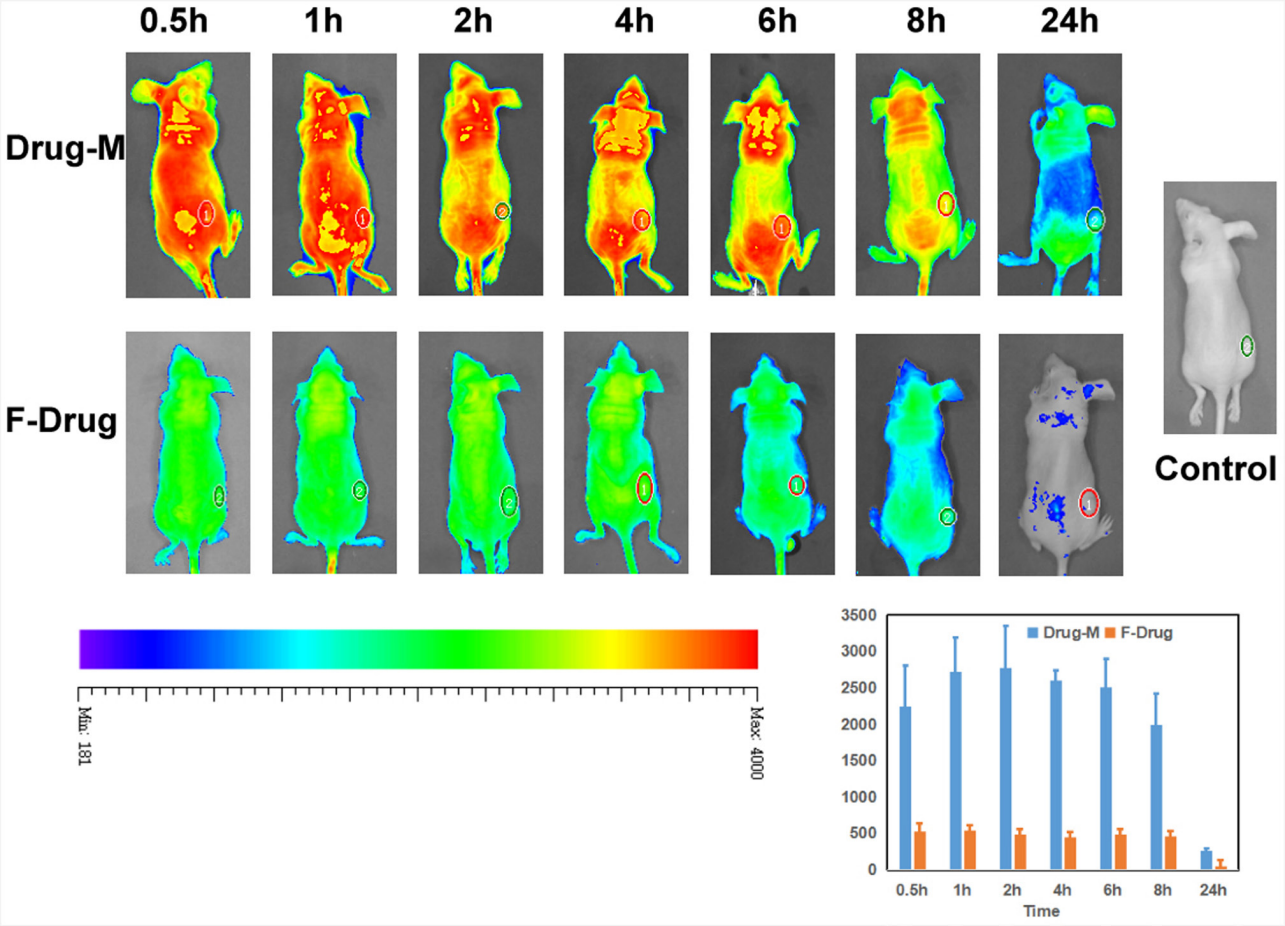
Imaging *in vivo* We analyzed the drug distribution of the free drug or drug nano micelles in the body by fluorescent markers. Nano-drug accumulation inside of the tumor was significantly more than free drug. And free drug in the body was removed very soon.

Mice bearing gliomas were injected with free drug or Drug/MPEG-PCL nano-micelles through the tail vein and imaged from the dorsal side at pre-determined time points using a bio-imaging system (Figure 12). Non-treated mice were used as the control group. The average fluorescent signal in the tumors of tumor-bearing mice injected with the drug/MPEG-PCL nano-micelles was higher than that of the tumor-bearing mice injected with free drug at the same time and same dose, which indicated that nano-micelles could enhanced more accumulation of drug in tumor.

## DISCUSSION

The anti-tumor effects of luteolin in various human cancers have been documented in previous published studies. However, few researches involve in checking the effectiveness of luteolin to suppress glioblastoma, which is charaterized by high proliferation, invasion and resistant to conventional chemotherapy. Recent study showed that luteolin effectively sensitized the cancer cells to several chemotherapeutic drugs by inhibiting Nrf2 activity [12]. In addition, luteolin has been reported to attenuate oxidative stress, brain edema and neurological deficits, which were common comorbidities in glioblastoma [22, 23]. Therefore, luteolin would become an ideal anti-glioma agent if it efficaciously block tumor growth or directly kill glioma cells.

Oral administration of luteolin is not reasonable due to its low bioavailability caused by the poor absorption and first pass effect. Recent research showed that the oral absorption rate of flavonoids is less than 10% and the maximum plasma concentration of luteolin was merely 3.79 μg/ml in rats which received a dose of 100 mg/kg orally [24, 25]. Moreover, luteolin is prone to be eliminated in circulation. Therefore, effective plasma concentration of luteolin applied either orally or by injection is hard to achieve.

Effective chemotherapy requires a continuously high local concentration of drugs in the tumor site. The diameter of Luteolin/MPEG-PCL micelles measured by DLS in our research was 36 nm. Since the interendothelial junctions of tumor mainly vary from 40 nm to 80 nm, luteolin/MPEG-PCL micelles were able to enter the tumor region through passive diffusion with little hindrance. Together with the impaired lymph drainage of nano-carriers in tumor, the enhanced penetration and retention (EPR) effect was greatly enchanced.

Another important aspect is the high stability of drug-loaded nanomaterials. Previous researches have demonstrated that PEGylated nanomaterials were able to avoid protein absorption in circulation, which is a key feature for drug delivery [26, 27]. Additionally, as the luteolin/MPEG-PCL micelles gradually degrades, encapsulated luteolin will be released slowly, which is determined by the core-shell structure of MPEG-PCL micelles. In this study, the result of drug release *in vitro* and pharmacokinetics in the xenograft nude mouse model suggested a more sustained release and prolonged circulation of Luteolin/MPEG-PCL compared with free luteolin. All these characteristics significantly contribute to the tumor accumulation of Luteolin/MPEG-PCL. With the help of MPEG-PCL micelles, phytochemical luteolin obtained higher bioavailability, improved tumor retention and lower off-target toxicity.

To evaluate the therapeutic efficacy of luteolin against GBM *in vivo*, U87 GBM transgenic zebra fish model, U87 GBM nude mice model and C6 nude mice model were employed. As shown in Figure 7 and Figure 8, Luteolin/MPEG-PCL micelles dramatically inhibited glioma growth in all animal models. Meanwhile, the Ki-67 expressions of glioma cells were detected by immunohistochemical staining. The treatment of Luteolin/MPEG-PCL micelles led to a markedly inhibition of cell proliferation. In order to explore the mechanism, tumor sections were stained with CD31 to examine the neovascularization of glioma. The microvessel density in the Luteolin/MPEG-PCL micelles group was much lower than other control groups, indicating that antiangiogenesis may play an important role in suppressing glioma by luteolin *in vivo*. Moreover, TUNEL assay was applied to evaluate cell apoptosis. In comparison with other groups, more apoptosis cells were significantly observed through electron microscope in group treated with Luteolin/MPEG-PCL micelles. The molecular mechanism of glioma cell apoptosis induced by Luteolin/MPEG-PCL micelles was further investigated in this research.

The effect of luteolin on the proliferation of glioma cells was observed *in vitro* with MTT and CFSE assay. It is evident that Luteolin/MPEG-PCL micelle suppresses glioma growth well *in vitro*. Also, a markedly decrease of phosphorylated p44/42 MAP kinase was found in the Luteolin/MPEG-PCL micelle group, which implies the p44/42 MAP kinase pathway may be involved in the tumor growth inhibition by luteolin. The phosphorylated p44/42 MAP kinase has been proved to promote proliferation of tumor cells [28].

Since some studies showed the anti-tumor effect of luteolin may be related to apoptosis, we employed FCM to determine the level of apoptosis and confirm the expression of proteins related to apoptosis, such as bcl-2, bax, caspase-9 and so forth [29–31]. The apoptosis induced by Luteolin/MPEG-PCL micelles was detected by FCM in our research. Meanwhile, as shown in Figure 5. , the expression of bcl-2 decreased significantly whereas markedly increases of bax, cleaved caspase-9 after Luteolin/MPEG-PCL micelles treatment for 48 hours. It suggests that Luteolin/MPEG-PCL micelle induces glioma apoptosis by the mitochondrial pathway.

In this research, we formulated Luteolin/MPEG-PCL micelles by a one step self-assembly method. The encapsulation of luteolin significantly improved its solubility and made it dispersible in water. Moreover, this drug delivery system ameliorated the pharmacokinetics. Together with the enhanced EPR effect, Luteolin/MPEG-PCL micelles prominently inhibited glioma growth *in vitro* and *vivo*. Luteolin suppresses tumor by inducing apoptosis via the mitochondrial pathway *in vitro*. RAS-RAF-MEK-MAPK pathway was involved in the cell growth inhibition by luteolin. The Luteolin/MPEG-PCL micelle has advantages of high anti-glioma activity, convenient manufacture and low toxicity. It has the potential of further clinical application in glioma chemotherapy.

## MATERIALS AND METHODS

### Materials

Luteolin and 3-(4,5-dimethylthiazol-2-yl)-2,5-diphenyl tetrazolium bromide (MTT) from Sigma (USA); Dulbecco’s Modified Eagle’s Medium (DMEM) and fetal bovine serum (FBS) from Gibco BRL (USA); methanol and acetic acid (HPLC grade) from Fisher Scientific (UK); and dimethyl sulfoxide (DMSO) and acetone from KeLong Chemicals (China). Antibodies purchased include: rat anti-mouse CD31 polyclonal antibody (BD PharmingenTM, USA), rabbit anti-mouse Ki67 antibody (Abcam, USA), and rhodamine-conjugated secondary antibody (Abcam, USA).

The MPEG (molecular weight = 2000) (Sigma–Aldrich, St. Louis, MO, USA) was dried in a one-necked flask under vacuum and magnetically stirred at 105 °C for 90 min before use.

MPEG (2000)-PCL(2000) diblock copolymer with a molecular weight of 4000 was synthesized by ring opening of ε-caprolactone, which was initiated by MPEG as descripting previously [21]. Briefly, MPEG and ε-CL were introduced into a dry glass ampoule under a nitrogen atmosphere. Sn(Oct)2 was then added into the reaction vessel under mild agitation, and the reaction system was kept at 130 °C for 6 h. The purified MPEG-PCL copolymer was kept in a desiccator before further use.

### Preparation of Luteolin/MPEG-PCL micelles

Luteolin/MPEG-PCL micelles were synthesized by a single-step self-assembly method. In brief, 6 mg luteolin and 94 mg MPEG-PCL diblock copolymer were dissolved in 2 ml acetone and added into 4 ml distilled water under constant stirring for 5 minutes. The above mixture was then evaporated under reduced pressure to remove acetone. The resultant formulation was called Luteolin/MPEG-PCL micelles solution. Finally, the prepared Luteolin/MPEG-PCL micelles were lyophilized and stored at 4°C.

### Characterization of Luteolin/MPEG-PCL micelles

We dissolved lyophilized Luteolin/MPEG-PCL micelles (10 mg) in methanol (0.1 ml) to determine the concentration of luteolin. The content of luteolin in the formulation was detected by high performance liquid chromatography (HPLC, Shimadzu LC-20AD, Japan). Drug loading (DL) and encapsulation efficiency (EE) of Luteolin/MPEG-PCL micelles were calculated by the following formulas (1) and (2):

$$\text{DL}=\frac{\text{Luteolin}}{\text{Polymer}+\text{Luteolin}}\times 100\%$$ (1)

$$\text{EE}=\frac{\text{Experimental Luteolin loading}}{\text{Theoretical Luteolin loading}}\times 100\%$$ (2)

Dynamic light scattering (DLS, Malvern Nano-ZS 90) was used to measure the particle size and zeta potential of Luteolin/MPEG-PCL micelles at 25 °C after previous dilution of samples with distilled water. This process was performed in triplicate.

Transmission electron microscopy (TEM, H-6009IV, Hitachi, Japan) was applied to determine the morphological characteristics of Luteolin/MPEG-PCL micelles. In brief, diluted suspension of Luteolin/MPEG-PCL micelles was placed drop-wise on a copper TEM grid. Then the grid was negatively stained with phosphotungsten acid (2%, w/v) for 20 minutes and allowed to dry. Thirty particles were observed to calculate the mean particle diameter.

The dialysis bag method was employed to evaluate the *in vitro* release kinetics of luteolin from Luteolin/MPEG-PCL micelles. Briefly, the dialysis bags (Sigma, St. Louis, MO, USA) which contained 1 ml of Luteolin/MPEG-PCL micelles were incubated in 200 ml phosphate buffer solution (PBS, pH 7.4) with 0.5% Tween-80 at 37 °C under gentle stirring. At different time points, 200 μl of the external medium was replaced with fresh medium. Then the luteolin content in samples were quantified by HPLC system. This process was repeated in triplicate.

### Pharmacokinetics

Pharmacokinetics investigation was conducted in Male Sprague-Dawley rats. The rats were randomly divided into two groups of five animals each. The two groups were injected with free luteolin (50 mg/kg) or Luteolin/MPEG-PCL micelles (50 mg/kg) respectively through the tail vein injection. At predetermined time points (predose, 15min, 30min, 1h, 2h, 4h, 8h, 12h, 24h), the blood was collected from carotid artery and serum concentration of luteolin was analyzed by HPLC. This process was repeated in triplicate.

### MTT test

The effects of free luteolin and Luteolin/MPEG-PCL micelles on C6 and U87 cell viability were evaluated by MTT method. In brief, C6 and U87 cells were seeded at a density of 5×10^3^ cells per well in 96-well plates and allowed to grow in DMEM added with 10% FBS for 24h. Cells were then treated with free luteolin or Luteolin/MPEG-PCL micelles at different concentrations respectively for 24h or 48h. At the end of incubation, fresh medium containing 500 μg/ml MTT was added. Thereafter cells were incubated for 3h at 37 °C. The formazan was then dissolved in DMSO during the process and the absorbance was measured at 570 nm using a plate reader (OPTImax, Molecular Dynamics). This process was repeated in triplicate.

### Cell proliferation assay

Cell proliferation was tested by CFSE staining method. U87 cell was stained with CellTrace™ CFSE staining solution and incubated for 20 minutes in a 37°C water bath. And U87 cells were seeded at density of 5×10^3^ cells per well in 6-well plates. The next day, cells were treated with free luteolin or Luteolin/MPEG-PCL micelles at different concentrations for 48h. After incubation cells were harvested and analyzed in a flow cytometry. This process was repeated in triplicate.

### Apoptosis study

Induction of apoptosis of U87 cells by free luteolin and Luteolin/MPEG-PCL micelles was investigated by flow cytometry (FCM, BD FACSCalibur). In brief, U87 cells were seeded at at density of 5×10^5^ cells per well in 6-well plates. The next day, cells were treated with free luteolin or Luteolin/MPEG-PCL micelles at different concentrations for 48h. After incubation cells were harvested and stained with 5 μl Annexin-V-FITC and 5 μl PI (Annexin V-FITC/PI Apoptosis Detection Kit). Then the apoptosis of stained cells were analyzed in a flow cytometry. This process was repeated in triplicate.

### Western blotting

Western blotting was performed to analyse the expression level of proteins involved in cell apoptosis and proliferation. U87 cells were seeded at a density of 5×10^4^ cells per plate and allowed to attach overnight. Cells were then treated with free luteolin, Luteolin/MPEG-PCL micelles at a concentration of 40 μg/ml, 20 μg/ml, 10 μg/ml, 5 μg/ml and EM for 48h. Cells treated with normal saline or blank micelles served as control groups. In the next step cells were lysed in RIPA (radioimmunoprecipitation assay) buffer. The protein extracts were separated by SDS-PAGE (sodium dodecyl sulfate-polyacrylamide) and transferred to PVDF (polyvinylidene fluoride) (Bio-Rad, Hercules, CA) membranes. The membranes were then probed with primary antibody against pro-caspase-9, cleaved-caspase-9, Bax, Bcl-2, MAPK, p-MAPK and β-actin followed by incubation with secondary antibodies. The blots were visualized by an enhanced chemiluminescence detection system (Amersham Biosciences Corp., Piscataway, NJ). This process was repeated in triplicate.

### Anti-glioma effect *in vivo*

The anti-glioma effects of Luteolin/MPEG-PCL micelles were studied in transgenic zebrafish model and nude mice xenograft model.

In the transgenic zebrafish model, U87 tumor cells were transfected with EGFP by lentiviral. Zebrafish embryos were stripped off egg sheath and anesthetized with 0.01% tricaine at 48 hpf. Under a Zeiss Stemi 2000-C dissecting microscope (Carl Zeiss Microimaging Inc., Thornwood, NY), zebrafish was then injected with 300 U87 tumor cells in perivitelline space using a Cell Tram Vario injector (Eppendorf, USA) with a glass micropipette (L = 50 mm, diameter of the needle opening about 25 μg/ml). At 24h after the implantation of tumor cells into zebrafish, Holtfreter’s solution (control), blank micelles, free luteolin and Luteolin/MPEG-PCL micelles were added into the incubating Holtfreter’s solution at a final luteolin concentration of 5 μg/ml. Finally, images of tumor were taken using a confocal microscope (DM6000 CS, Leica, Germany) at 5 days post-inoculation. This process was repeated in triplicate.

In the nude mice xenograft model, nude mice were injected subcutaneously with 0.1 mL of C6 or U87 cells containing 1×10^7^ cells in the right flank. As the tumor grows to 0.1 cm^3^, the mice were randomly assigned to 4 groups and treated with normal saline (NS), empty micelles (EM), free luteolin (FL) (50 mg/kg body weight) or Luteolin/MPEG-PCL (LM) (50 mg/kg body weight) micelles everyday respectively. The diameter of tumor was assessed by caliper in two dimensions every other day and the body-weight of mice was measured in the meanwhile. Tumor volume was calculated according to the following formula: V=0.52*(ab^2^), where a represented the length while b represented the width. Mice were sacrificed at predetermined time and tumors were immediately weighed. This process was repeated in triplicate.

### TUNEL assay

Apoptosis of U87 tumors induced by Luteolin/MPEG-PCL micelles was detected by TUNEL staining. When tumor tissues were sectioned, a TUNEL kit (Promega, Madison, WI, USA) was used to analyze apoptotic cells within tumors following the manufacturer's protocol. Five equal-sized tumor sections were detected. TUNEL positive cells were observed under a fluorescent microscope (×400).

### Immunohistochemical examination of Ki67 and CD31

Tumor proliferation activity and neovascularization in tumor tissues were investigated by Immunohistochemical analysis with antibody Ki67 and CD31. Tumor sections were stained with rabbit anti-mouse ki67 polyclonal antibody (1: 50; BD PharmingenTM, USA) or rabbit anti-mouse CD31. Then they were washed twice with PBS, and incubated with FITC- or Rhodamine-conjugated secondary antibody respectively for 1h (Abcam, USA). Ki-67 positive and total cells were counted in each tumor section under microscope and Ki-67 labeling index was calculated. The CD31 positive microvessels were counted under high power field (×400).

### Imaging *in vivo*

For imaging *in vivo* animal experiments, 6-week-old female nude BALB/c mice were used. Glioma tumors were induced in mice by inoculating them in the back with U87 cells (1.0 ×10^7^). When the tumor volume reached approximately 150–200 mm^3^, Coumarin/MPEG-PCL micelles or free coumarin (1 mg/kg) were intravenously injected through the tail vein. After i.v. injection of the drug, optical fluorescence imaging was performed by positioning each mouse on an animal plate in the *vivo* imaging system that was heated to 37 °C (Quick View 3000). The images were obtained 0.5, 1, 2, 4, 6, 8 and 24h after injection of the test drugs.

### Statistical analysis

Results were expressed as mean ± SD. Statistical analysis was performed with one-way analysis of variance (ANOVA) using SPSS 15.0 software (Chicago, IL, USA). Values of P < 0.05 are indicative of statistically significant.

## References

[1] Chen J, McKay RM, Parada LF (2012). Malignant glioma: lessons from genomics, mouse models, and stem cells. Cell.

[2] Brower V (2015). MRI study identifies three subtypes of glioblastoma. Lancet Oncol.

[3] Venneti S, Dunphy MP, Zhang H, Pitter KL, Zanzonico P, Campos C, Carlin SD, La Rocca G, Lyashchenko S, Ploessl K, Rohle D, Omuro AM, Cross JR (2015). Glutamine-based PET imaging facilitates enhanced metabolic evaluation of gliomas *in vivo*. Sci Transl Med.

[4] Preusser M, Lim M, Hafler DA, Reardon DA, Sampson JH (2015). Prospects of immune checkpoint modulators in the treatment of glioblastoma. Nat Rev Neurol.

[5] Yang HW, Lu YJ, Lin KJ, Hsu SC, Huang CY, She SH, Liu HL, Lin CW, Xiao MC, Wey SP, Chen PY, Yen TC, Wei KC (2013). EGRF conjugated PEGylated nanographene oxide for targeted chemotherapy and photothermal therapy. Biomaterials.

[6] Fan QW, Cheng C, Hackett C, Feldman M, Houseman BT, Nicolaides T, Haas-Kogan D, James CD, Oakes SA, Debnath J, Shokat KM, Weiss WA (2010). Akt and autophagy cooperate to promote survival of drug-resistant glioma. Sci Signal.

[7] Godlewski J, Nowicki MO, Bronisz A, Nuovo G, Palatini J, De Lay M, Van Brocklyn J, Ostrowski MC, Chiocca EA, Lawler SE (2010). MicroRNA-451 regulates LKB1/AMPK signaling and allows adaptation to metabolic stress in glioma cells. Mol Cell.

[8] Yang C, Sudderth J, Dang T, Bachoo RM, McDonald JG, DeBerardinis RJ (2009). Glioblastoma cells require glutamate dehydrogenase to survive impairments of glucose metabolism or Akt signaling. Cancer Res.

[9] Mashima T, Sato S, Sugimoto Y, Tsuruo T, Seimiya H (2009). Promotion of glioma cell survival by acyl-CoA synthetase 5 under extracellular acidosis conditions. Oncogene.

[10] Behbahani M, Zadeh MS, Mohabatkar H (2013). Evaluation of antiherpetic activity of crude extract and fractions of Avicenna marina, *in vitro*. Antiviral Res.

[11] Paterniti I, Impellizzeri D, Di Paola R, Navarra M, Cuzzocrea S, Esposito E (2013). A new co-ultramicronized composite including palmitoylethanolamide and luteolin to prevent neuroinflammation in spinal cord injury. J Neuroinflammation.

[12] Tang X, Wang H, Fan L, Wu X, Xin A, Ren H, Wang XJ (2011). Luteolin inhibits Nrf2 leading to negative regulation of the Nrf2/ARE pathway and sensitization of human lung carcinoma A549 cells to therapeutic drugs. Free Radic Biol Med.

[13] Polier G, Giaisi M, Köhler R, Müller WW, Lutz C, Buss EC, Krammer PH, Li-Weber M (2015). Targeting CDK9 by wogonin and related natural flavones potentiates the anti-cancer efficacy of the Bcl-2 family inhibitor ABT-263. Int J Cancer.

[14] Byun S, Lee KW, Jung SK, Lee EJ, Hwang MK, Lim SH, Bode AM, Lee HJ, Dong Z (2010). Luteolin inhibits protein kinase C(epsilon) and c-Src activities and UVB-induced skin cancer. Cancer Res.

[15] Kwon EY, Jung UJ, Park T, Yun JW, Choi MS (2015). Luteolin attenuates hepatic steatosis and insulin resistance through the interplay between the liver and adipose tissue in mice with diet-induced obesity. Diabetes.

[16] Xu N, Zhang L, Dong J, Zhang X, Chen YG, Bao B, Liu J (2014). Low-dose diet supplement of a natural flavonoid, luteolin, ameliorates diet-induced obesity and insulin resistance in mice. Mol Nutr Food Res.

[17] Wang H, Liu K, Geng M, Gao P, Wu X, Hai Y, Li Y, Li Y, Luo L, Hayes JD, Wang XJ, Tang X (2013). RXRα inhibits the NRF2-ARE signaling pathway through a direct interaction with the Neh7 domain of NRF2. Cancer Res.

[18] Zaytseva YY, Rychahou PG, Gulhati P, Elliott VA, Mustain WC, O'Connor K, Morris AJ, Sunkara M, Weiss HL, Lee EY, Evers BM (2012). Inhibition of fatty acid synthase attenuates CD44-associated signaling and reduces metastasis in colorectal cancer. Cancer Res.

[19] Tai Z, Lin Y, He Y, Huang J, Guo J, Yang L, Zhang G, Wang F (2014). Luteolin sensitizes the antiproliferative effect of interferon α/β by activation of Janus kinase/signal transducer and activator of transcription pathway signaling through protein kinase A-mediated inhibition of protein tyrosine phosphatase SHP-2 in cancer cells. Cell Signal.

[20] Xie F, Lang Q, Zhou M, Zhang H, Zhang Z, Zhang Y, Wan B, Huang Q, Yu L (2012). The dietary flavonoid luteolin inhibits Aurora B kinase activity and blocks proliferation of cancer cells. Eur J Pharm Sci.

[21] Gao X, Wang S, Wang B, Deng S, Liu X, Zhang X, Luo L, Fan R, Xiang M, You C, Wei Y, Qian Z, Guo G (2015). Improving the anti-ovarian cancer activity of docetaxel with biodegradable self-assembly micelles through various evaluations. Biomaterials.

[22] Zhou F, Qu L, Lv K, Chen H, Liu J, Liu X, Li Y, Sun X (2011). Luteolin protects against reactive oxygen species-mediated cell death induced by zinc toxicity via the PI3K-Akt-NF-κB-ERK-dependent pathway. J Neurosci Res.

[23] Xu J, Wang H, Ding K, Zhang L, Wang C, Li T, Wei W, Lu X (2014). Luteolin provides neuroprotection in models of traumatic brain injury via the Nrf2-ARE pathway. Free Radic Biol Med.

[24] Thilakarathna SH, Rupasinghe HP (2013). Flavonoid bioavailability and attempts for bioavailability enhancement. Nutrients.

[25] Lin LC, Pai YF, Tsai TH (2015). Isolation of luteolin and luteolin-7-O-glucoside from Dendranthema morifolium Ramat Tzvel and their pharmacokinetics in rats. J Agric Food Chem.

[26] Xu H, Fan M, Elhissi AM, Zhang Z, Wan KW, Ahmed W, Phoenix DA, Sun X (2015). PEGylated graphene oxide for tumor-targeted delivery of paclitaxel. Nanomedicine (Lond).

[27] Zhang Y, Velasco O, Zhang X, Ting K, Soo C, Wu BM (2014). Bioactivity and circulation time of PEGylated NELL-1 in mice and the potential for osteoporosis therapy. Biomaterials.

[28] Mohankumar KM, Xu XQ, Zhu T, Kannan N, Miller LD, Liu ET, Gluckman PD, Sukumar S, Emerald BS, Lobie PE (2007). HOXA1-stimulated oncogenicity is mediated by selective upregulation of components of the p44/42 MAP kinase pathway in human mammary carcinoma cells. Oncogene.

[29] Lucas CD, Allen KC, Dorward DA, Hoodless LJ, Melrose LA, Marwick JA, Tucker CS, Haslett C, Duffin R, Rossi AG (2013). Flavones induce neutrophil apoptosis by down-regulation of Mcl-1 via a proteasomal-dependent pathway. FASEB J.

[30] Domitrović R, Cvijanović O, Pugel EP, Zagorac GB, Mahmutefendić H, Škoda M (2013). Luteolin ameliorates cisplatin-induced nephrotoxicity in mice through inhibition of platinum accumulation, inflammation and apoptosis in the kidney. Toxicology.

[31] Ding S, Hu A, Hu Y, Ma J, Weng P, Dai J (2014). Anti-hepatoma cells function of luteolin through inducing apoptosis and cell cycle arrest. Tumour Biol.

